# Anti-TB treatment outcomes in TB meningitis: A systematic review and meta-analysis

**DOI:** 10.1016/j.nmni.2025.101623

**Published:** 2025-08-21

**Authors:** Samin Afazel, Mohammad J. Nasiri, Vishwanath Venketaraman

**Affiliations:** a Department of Microbiology, School of Medicine, Shahid Beheshti University of Medical Sciences, Tehran, Iran; b College of Osteopathic Medicine of the Pacific, Western University of Health Sciences, Pomona, CA, 91766-1854, USA

**Keywords:** Tuberculous meningitis, Mortality, Loss to follow-up, Neurological disability, HIV, Modified rankin scale, Barthel index, Systematic review, Meta-analysis, Anti-TB drugs, Treatment outcomes

## Abstract

**Introduction:**

Tuberculous meningitis (TBM) remains a leading cause of mortality and neurological disability in both children and adults. This systematic review and meta-analysis aim to assess the treatment outcomes of anti-tuberculosis drugs in TBM patients, focusing on mortality and neurological disability.

**Methods:**

We conducted a comprehensive literature search on PubMed/MEDLINE, EMBASE, and Cochrane CENTRAL databases to identify articles reporting treatment outcomes in TBM up to December 15, 2024. Studies included in the analysis reported treatment outcomes for TBM patients. Pooled analyses were performed using random-effects model to assess mortality rates, neurological disability, and loss to follow-up.

**Results:**

A total of 10 studies involving 2005 patients were included in the analysis. The pooled all-cause mortality rate across studies was 27.7 % (95 % CI: 22.6–33.4 %, *I*^*2*^: 76 %), with higher mortality observed in HIV-positive individuals (40.3 %) compared to HIV-negative patients (17.1 %). The pooled rate of loss to follow-up was 6.6 % (95 % CI: 4.7–9.1 %). Subgroup analysis revealed that the mortality rate increased from 18.9 % at 3 months to 29.1 % at 6 months. The frequency of neurological disability was higher among studies using the Modified Rankin Scale (41.7 %) compared to the Barthel Index (14.1 %).

**Conclusions:**

This study highlights the high mortality and significant neurological disability in TBM patients, particularly in HIV-positive individuals. Our findings emphasize the need for standardized outcome reporting and the incorporation of new therapeutic strategies, and improved diagnostic tools, to enhance clinical outcomes. Future research should focus on addressing these areas to optimize treatment protocols and reduce the burden of TBM.

## 1 Introduction

Tuberculous meningitis (TBM), a severe manifestation of central nervous system tuberculosis (TB), remains a leading cause of morbidity and mortality among individuals with TB [1,2]. The World Health Organization (WHO) estimates that in 2023, approximately 10.8 million people developed TB globally, with an estimated 1.25 million deaths. Among these, TBM is responsible for significant clinical challenges, with an estimated 1–5 % of all TB patients suffering from this life-threatening condition. The global burden of TBM continues to be a major public health concern, especially given the rising incidence of drug-resistant TB [[3], [4], [5]].

Effective treatment of TBM relies on a combination of standard anti-TB therapies, but the optimal therapeutic regimen and outcomes remain subjects of ongoing investigation [6]. While first-line anti-TB therapy (ATT), is commonly used, its effectiveness in TBM is frequently compromised by poor penetration of drugs into the cerebrospinal fluid (CSF), slow response to treatment, and complications related to drug resistance [7]. Moreover, the advent of HIV and the increasing number of co-infected patients have further complicated the treatment landscape for TBM, requiring tailored therapeutic strategies to address both infections concurrently [8,9].

This systematic review and meta-analysis aim to provide a comprehensive evaluation of the treatment outcomes of TBM patients, focusing on the efficacy of current anti-TB regimens.

## 2 Methods

This systematic review was conducted in accordance with the Preferred Reporting Items for Systematic Reviews and Meta-Analyses (PRISMA) guidelines [10] (PROSPERO ID: CRD42024618814).

### 2.1 Search strategy

A systematic electronic search was performed on PubMed/MEDLINE, EMBASE and Cochrane CNTERAL databases to identify articles reporting treatment outcomes in TBM, covering the period up to December 15, 2024. The search utilized a combination of controlled vocabulary terms (e.g., Medical Subject Headings [MeSH] terms) and free-text keywords to capture relevant studies. Key search terms included, but were not limited to, “tuberculous meningitis”, “treatment outcomes” and “anti-tuberculosis therapy”. Reference lists of relevant articles were also manually checked for additional studies.

### 2.2 Selection criteria

Eligibility for inclusion in this review was determined using the Population, Intervention, Comparison, Outcome, and Study Design (PICOS) framework [11]:

• Population: Patients with confirmed or suspected TBM.
• Intervention: Anti-TB therapy
• Outcomes: All-cause mortality, loss to follow-up (due to treatment abandonment or other reasons), neurological sequelae, as reported by each study, with disability assessed using the Modified Rankin Scale (mRS) (>2) or Barthel Index (BI) (<80) or without specified scale
• Study Design: Randomized controlled trials (RCTs)
• Studies were excluded if they were case reports, case series, cohorts, editorials, narrative reviews, or studies focusing on specific complications (e.g., hydrocephalus, tuberculomas) rather than TBM treatment outcomes. Studies without a standard anti-TB drug regimen, non-English publications, were also excluded. Duplicate studies were excluded in favor of the one with the largest sample size.

### 2.3 Review process

All identified articles were uploaded to EndNote. Duplicates were removed manually. Two reviewers (SA, MJN) independently screened titles and abstracts of the articles, and any disagreement was resolved by a third reviewer (VV). Subsequently, they evaluated full texts of all potentially eligible studies and any disagreement was resolved by the third reviewer.

### 2.4 Data extraction

Data were independently extracted by two authors using a standardized abstraction form, with any discrepancies resolved through discussion with a third author. The following data were collected from each included study: treatment outcomes, patient demographics, study design characteristics, and relevant clinical variables. Possible, probable or definite TBM status was assigned based on Consensus Research Definition [12]. The primary outcomes of interest included all-cause mortality, neurological sequelae, and treatment abandonment or loss to follow-up. Neurological sequelae were defined as any impairment in a patient's ability to perform tasks that were previously achievable, as assessed by the mRS (score >2) or the BI (score <80) or even without a specified scale [13,14].

### 2.5 Quality assessment

The quality of included studies was assessed using the Cochrane Risk of Bias tool, which evaluates several key factors, including random sequence generation, allocation concealment, blinding of participants and personnel, blinding of outcome assessors, completeness of outcome data, and selective reporting [15]. Studies were categorized as having a low risk of bias if no concerns were identified, a high risk of bias if significant concerns were present, or an unclear risk of bias if there was insufficient information to make a definitive judgment.

### 2.6 Data analysis

The Comprehensive Meta-Analysis software, version 3.0 (Biostat Inc., Englewood, NJ, USA), was used for statistical computations [16]. Point estimates and 95 % confidence intervals (CIs) for the proportion of patients achieving treatment outcomes were calculated. A random-effects or fixed-effects model was chosen based on the heterogeneity of the effect sizes [17]. The assessment of between-study heterogeneity was conducted using Cochran's Q test and the *I*^*2*^ statistic [18]. Furthermore, publication bias was statistically evaluated using Egger's test and Begg's test, with a P-value <0.05 considered statistically significant publication bias [19].

## 3 Results

**Fig. 1:**
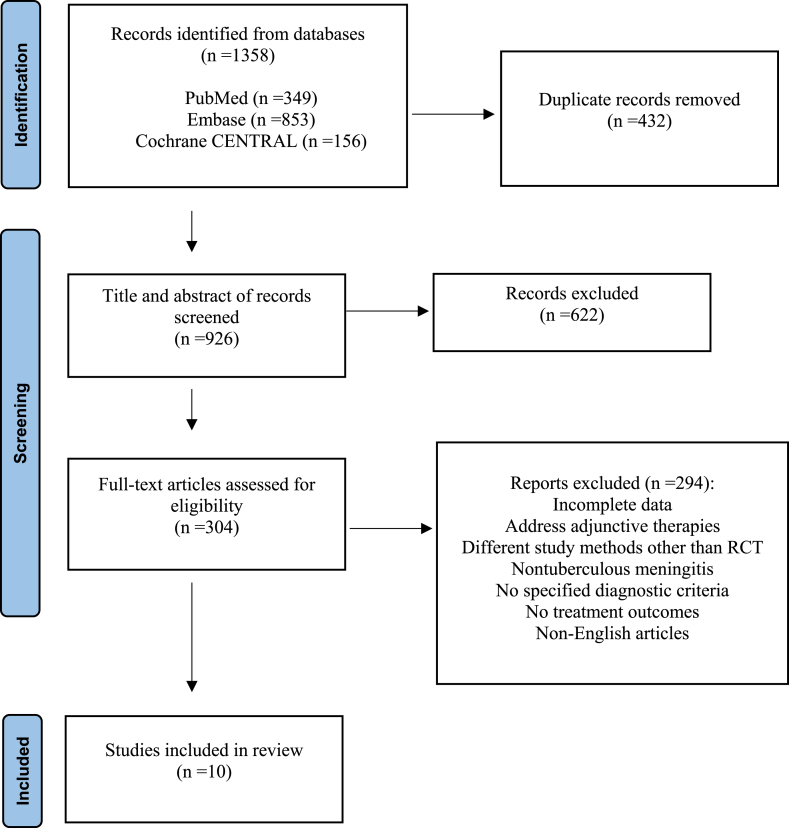
Flow chart of study selection.

Fig. 1 presents a flowchart illustrating the literature search process. The initial database search yielded 1358 publications. After removing duplicates, 926 articles were screened by title and abstract. Of these, 304 were selected for full-text review, resulting in the inclusion of 10 studies [[20], [21], [22], [23], [24], [25], [26], [27], [28], [29]]. The key characteristics of the included studies are summarized in Table 1.

**Table 1:** Study characteristics.

First Author's Name	Year of Publication	Study Design	Country	Number of patients	Mean age (Year)	Male	No. of HIV (%)	Regimen (Cases)	Regimen (Controls)	Diagnosis Method	Follow-up Time	Intensive Phase (Treatment)	Intensive Phase (Duration)	Continuation Phase (Treatment)	Continuation Phase (Duration)
Sahib	2023	Pilot study	India	29	25	48 %	0	Standard ATT + 600 mg BD Linezolid	Standard ATT	Clinical, CSF, and radiological findings	3 months	HRZE/S + Lzd	28 days	HRZE/S	3 months
Davis	2023	Phase 2A	South Africa	52	39	62.5 %	52 (100 %)	High-dose rifampicin + linezolid + aspirin	Standard ATT	Clinical, CSF, and imaging findings	6 months	High-dose rifampicin, linezolid, aspirin	56 days	Standard ATT	7 months
Paradkar	2022	Phase II	India-Malawi	37	6	49 %	0	High-dose rifampicin regimens (HR30ZE, HR30ZL)	SOC per WHO (HR15ZE)	CT, MRI, and CSF lab tests, TBM status by Consensus Definition	52 weeks	HR30ZE/HR30ZL/HR15ZE	8 weeks	HR15ZE	12 months
Cresswell	2021	Phase 2, Open-label RCT	Uganda	61	35	55.7 %	56 (91.8 %)	High-dose oral and IV rifampicin	Standard-dose ATT	Clinical and CSF findings	24 weeks	High-dose rifampicin (PO-35, IV-20)	8 weeks	Standard ATT	9–12 months
Misra	2021	Randomized Controlled Trial	India	80	26	46 %	0	Sequential ATT	Standard ATT	Clinical, MRI, CSF	6 months	Sequential treatment (R10, H, Z, E)	6 months	Standard ATT	–
Thuong	2019	Randomized Controlled Trial	Vietnam	692	35 (29–46)	68.6 %	288 (41.6 %)	Intensified ATT with high-dose rifampicin	Standard ATT	GeneXpert, clinical findings	9 months	Intensified rifampicin, levofloxacin	8 weeks	Standard ATT	9 months
Heemskerk	2016	Randomized, double-blind, placebo-controlled	Vietnam	817	35 (29–46)	68.5 %	349 (42.7 %)	Higher-dose rifampin and levofloxacin	Standard ATT	Clinical, CSF findings	9 months	Rifampin (15 mg/kg) + Levofloxacin	8 weeks	Standard ATT	7 months
Kalita (2016)	2016	Open-label, randomized controlled trial	India	57	35 (median)	52.6 %	NM (0 in base study)	RHZE + levofloxacin	RHZE	Clinical, CSF, and MRI findings	6 months	RHZE + Levofloxacin	8 weeks	RHZE	6 months
Kalita (2014)	2014	Open-label, randomized controlled trial	India	120	34.5	55.8 %	4 (3 in base study) (3 %)	Levofloxacin	Rifampicin	Clinical, MRI, and CSF findings	6 months	RHZE or Levofloxacin	8 weeks	Standard ATT	4 months
Ruslami	2013	Phase 2, Open-label RCT	Indonesia	60	28 (16–64)	55 %	7 (11.6 %)	High-dose rifampicin and moxifloxacin	Standard-dose ATT	CSF findings, microbiology	6 months	High-dose rifampicin, moxifloxacin	14 days	Standard ATT	4 months

A total of 2005 patients from 10 studies, including pilot studies, phase II trials, RCTs, and open-label RCTs, were included in this systematic review and meta-analysis. The studies, conducted across India, South Africa, Uganda, Vietnam, Malawi, and Indonesia, which are all currently on the WHO list of countries with high TB burden [3], had varying sample sizes, ranging from 29 to 817 participants, with a mean age between 6 and 39 years. Male patients constituted 46 %–68.6 % of the populations, and the proportion of HIV-positive patients ranged from 0 % to 100 %. Most studies utilized standard ATT as the control, while experimental regimens included high-dose rifampicin, linezolid, moxifloxacin, and levofloxacin. Diagnosis of TBM was based on clinical, CSF, radiological, and microbiological findings. The follow-up periods varied, from 3 months to 12 months. Treatment regimens included intensive phases lasting 28 days to 8 weeks, with some studies assessing extended or alternative continuation phases up to 12 months (Table 1).

### 3.1 Quality assessment

The quality assessment of the included studies, based on the Cochrane tool, is summarized in Table 2. Most studies were rated as low risk for random sequence generation and allocation concealment. However, three studies (Cresswell, Kalita 2014, and Ruslami) had a high risk of incomplete outcome data. Despite these issues, the overall study quality was acceptable, although blinding bias may have influenced some outcomes.

**Table 2:** Quality assessment (the Cochrane tool).

Author	Random sequence generation	Allocation concealment	Blinding of participants and personnel	Blinding of outcome assessment	Incomplete outcome data	Selective reporting	Other bias
Sahib	Low risk	Low risk	High risk	Low risk	Low risk	Low risk	Low risk
Davis	Low risk	Low risk	High risk	Low risk	Low risk	Low risk	Low risk
Paradkar	Low risk	Low risk	High risk	Low risk	Low risk	Low risk	Low risk
Cresswell	Low risk	Low risk	High risk	Low risk	High risk	Low risk	Low risk
Misra	Low risk	Low risk	High risk	Low risk	Low risk	Low risk	Low risk
Thuong	Low risk	Low risk	High risk	Low risk	Low risk	Low risk	Low risk
Heemskerk	Low risk	Low risk	High risk	Low risk	Low risk	Low risk	Low risk
Kalita (2016)	Low risk	Low risk	High risk	Low risk	Low risk	Low risk	Low risk
Kalita (2014)	Low risk	Low risk	High risk	Low risk	High risk	Low risk	Low risk
Ruslami	Low risk	Low risk	High risk	Low risk	High risk	Low risk	Low risk

### 3.2 Clinical profiles and treatment outcomes

The clinical profiles and treatment outcomes of the included studies are summarized in Table 3. The proportion of patients diagnosed with definite TBM ranged from 20 % (24 out of 120) to 57.4 % (397 out of 692) across studies. For probable TBM, this ranged from 23 % (14 out of 61) to 68.4 % (39 out of 57), while possible TBM diagnoses varied from none to 40.3 % (21 out of 52). Nervous system disorders were reported in several studies, with 18–103 patients (up to 66.6 %) exhibiting severe neurological impairments, as assessed using the mRS, with scores ranging from 3 to 6. Loss to follow-up varied, with the highest rate being 13.4 %, and the lowest rate at 0 %. Mortality rates varied significantly across studies, ranging from 2.7 % to 50 %. Among HIV-positive participants, mortality rates were as high as 58.5 % in some studies, while others reported lower rates, such as 15.5 %. The proportion of HIV-positive patients varied widely, from 0 % to 100 % of the total study populations. These findings highlight the substantial burden of mortality and neurological impairment in TBM, particularly among HIV-positive individuals, underscoring the urgent need for early diagnosis and effective treatment interventions.

**Table 3:** Clinical profiles and treatment outcomes.

First Author's Name	Number of patients	Number of Patients Receiving Corticosteroids	Number of Definite TBM	Number of Probable TBM	Number of Possible TBM	Number of Nervous Systems Disorders	Number of Loss to Follow-up	Number of Mortality	Number of Mortality Among HIV
Sahib	29	29	15	12	NR	18/27 (mRS 3–6)	2	6/27	–
Davis	52	52	17	13	21	14/50 (mRS 4–6)	7	7/45	7/45
Paradkar	37	37	21	10	6	8	NR	1	–
Cresswell	61	61	31	14	12	16/58	3	24/58	NR
Misra	80	80	31	NR	NR	27/77 (mRS>2)	3	13/77	–
Thuong	692	692	397	170	125	103/692	NM	192/692	103/176
Heemskerk	817	817	407	214	174	81/817	53	227/817	136/349
Kalita (2016)	57	57	18	39	0	7/56 (BI)	1	11/56	–
Kalita (2014)	120	120	24	36	NR	15/100 (BI)	NR	34/100	NR
Ruslami	60	60	31	20	9	NR	0	30/60	4/7

NR: Not reported.

### 3.3 Mortality and lost to follow-up

**Fig. 2:**
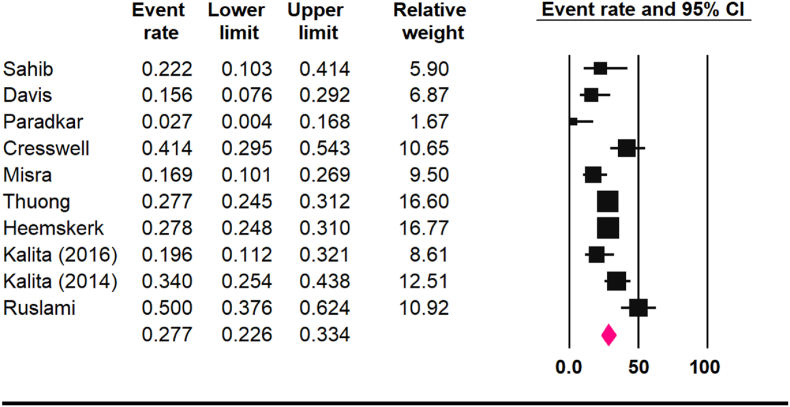
The pooled all-cause mortality rate.

The pooled all-cause mortality rate across the included studies was 27.7 % (95 % CI: 22.6–33.4 %, *I*^*2*^: 76 %) (Fig. 2), with no evidence of publication bias (Begg test: p = 0.1, Egger's test: *P* = 0.5). The pooled rate of loss to follow-up was 6.6 % (95 % CI: 4.7–9.1 %, *I*^*2*^: 13.0 %), with no evidence of publication bias (*P* = 0.7).

### 3.4 Subgroup analysis

#### 3.4.1 Mortality rates

Subgroup analyses based on follow-up time revealed that the pooled mortality rate at 3 months was 18.9 % (95 % CI: 11.8–28.8 %) with high heterogeneity (*I*^*2*^ = 75 %, *P* < 0.01) (Table 4). At 6 months, the pooled mortality rate increased to 29.1 % (95 % CI: 18.8–42.1 %) with even higher heterogeneity (*I*^*2*^ = 81 %, *P* < 0.01) (Table 4). Additionally, subgroups based on HIV status showed significant differences in mortality rates. HIV-positive patients had a pooled mortality rate of 40.3 % (95 % CI: 24.7–58.2 %), with very high heterogeneity (*I*^*2*^ = 90 %, *P* < 0.01) (Table 4). In contrast, HIV-negative patients had a significantly lower pooled mortality rate of 17.1 % (95 % CI: 10.9–25.8 %) with moderate heterogeneity (*I*^*2*^ = 35 %, *P* < 0.01) (Table 4).

**Table 4:** Subgroup analysis of mortality rates based on follow-up time and HIV status.

Subgroups	Variables	No. of study	Pooled frequency	CI 95 %	I^2^, *P* value	Begg *P* value
Follow-up Time (Mortality Rates)	3 months	6	18.9	11.8–28.8	75 %, *P<0.01*	0.2
	6 months	6	29.1	18.8–42.1	81 %, *P<0.01*	0.1
HIV	Positive	4	40.3	24.7–58.2	90 %, *P<0.01*	1
	Negative	4	17.1	10.9–25.8	35 %, *P<0.01*	1

#### 3.4.2 Neurological disability

Subgroup analysis of neurological disability revealed a higher pooled frequency of disability among studies using the mRS, with a pooled frequency of 41.7 % (95 % CI: 23.8–62 %) and high heterogeneity (*I*^*2*^ = 81 %, *P* < 0.01) (Table 5). In studies using the BI, the pooled frequency of neurological disability was lower at 14.1 % (95 % CI: 9.5–20.6 %), with no reported heterogeneity due to the inclusion of only two studies in this category. (Table 5). In studies without a specified assessment scale, the pooled frequency of neurological disability was 16.5 % (95 % CI: 10.9–24.2 %) and high heterogeneity (*I*^*2*^ = 86 %, *P* < 0.01) (Table 5).

**Table 5:** Subgroup analysis of neurological disability based on assessment scales.

Subgroups	Variables	No. of study	Pooled frequency	CI 95 %	I2, *P* value	Begg *P* value
Neurological disability	MRS	3	41.7	23.8–62	81 %, *P<0.01*	0.7
	BI	2	14.1	9.5–20.6	–	–
	Without specified scale	4	16.5	10.9–24.2	86 %, *P<0.01*	1

## 4 Discussion

This meta-analysis found an all-cause mortality rate of 27.7 % in TBM patients, rising from 18.9 % at 3 months to 29.1 % at 6 months. HIV-positive patients had a significantly higher mortality rate (40.3 %) compared to HIV-negative patients (17.1 %), highlighting the severe impact of HIV on TBM outcomes. Additionally, 41.7 % of survivors exhibited long-term neurological impairment (mRS).

Our study differs from those by Stadelman et al. [30] and Wang et al. [31] in several key ways. Stadelman et al. reported a 6-month mortality rate of 23 %, with HIV-positive patients showing a much higher mortality rate (57 %) than HIV-negative patients (16 %). Wang et al. found a mortality rate of 24.7 %, with increased risk in patients with stage III TB or HIV co-infection. Both studies included case series, cohorts, and RCTs, while our analysis focused solely on RCTs, providing higher-quality evidence. Moreover, unlike prior studies that focused on patients aged ≥15 years, we included all age groups, acknowledging that TBM commonly affects young children. Another important distinction is that our study strictly examined anti-TB drugs without adjunctive therapies (treatments such as corticosteroids and other supportive therapies used alongside standard anti-TB drugs), avoiding potential bias and providing a clearer assessment of drug efficacy. Additionally, our use of the most up-to-date data offers a current perspective on treatment outcomes, particularly in light of evolving TBM treatment strategies. This focus adds novelty and value to our findings.

The clinical implications of our study highlight the urgent need for more effective treatment strategies, particularly given the high mortality rates in TBM even with anti-TB drug regimens. New drugs, such as bedaquiline and delamanid, have shown promise in drug-resistant TB and could potentially enhance outcomes in TBM when integrated into existing regimens [32,33]. Additionally, adjunctive therapies like glutathione, which have been explored for their immune-modulating and antioxidant properties, might play a critical role in reducing CNS inflammation and improving drug delivery to the brain, addressing challenges in TBM management [[34], [35], [36], [37]].

In addition to these treatment advances, the role of molecular diagnostic tools, such as the GeneXpert MTB/RIF and Xpert MTB/RIF Ultra assays, cannot be overlooked. These tools have significantly improved the early detection of TBM by offering rapid and specific identification of *Mycobacterium tuberculosis* in CSF [[38], [39], [40]]. Xpert Ultra, in particular, demonstrates enhanced sensitivity, especially in HIV-associated TBM cases [41]. Combining these molecular diagnostics with advanced imaging techniques, such as MRI and CT, enables earlier diagnosis and more personalized treatment approaches, potentially reducing morbidity and mortality in TBM patients [42,43].

Our findings highlight the substantial burden of mortality and neurological disability in TB meningitis; however, the limited availability of long-term follow-up data restricts a comprehensive understanding of the disease's chronic impact and the sustained effectiveness of treatment regimens. Long-term outcome assessment is critical to capture delayed neurological sequelae, treatment-related complications, and quality of life measures that may not be evident in short-term studies. Future research should prioritize extended follow-up periods and standardized reporting of long-term clinical, functional, and cognitive outcomes to better inform prognosis and optimize patient management. Incorporating long-term data will also facilitate evaluation of emerging therapies and adjunctive interventions, ultimately improving care strategies for this high-risk population.

Our study has some limitations. Significant heterogeneity across studies, particularly in mortality and neurological disability rates, limits the generalizability of our findings and highlights the need for more standardized studies. Additionally, the lack of uniformity in measuring neurological disability and limited long-term follow-up data restricts our understanding of long-term treatment effects. Moreover, many included studies did not report outcomes stratified by TBM diagnostic certainty (definite vs. probable/possible) or by clinical severity, and definitions varied widely. This precluded subgroup analyses based on these important clinical factors.

## 5 Conclusions

This study provides a comprehensive overview of treatment outcomes in TBM, highlighting the high mortality rates and significant neurological disability among survivors. While focusing on anti-TB drug regimens, our results emphasize the importance of incorporating new therapeutic strategies, including novel drug regimens, adjunctive therapies, and diagnostic advancements, to improve clinical outcomes in this challenging disease. Future research should address these gaps to optimize treatment protocols and reduce the burden of TBM.

## CRediT authorship contribution statement

**Samin Afazel:** Supervision, Investigation, Data curation. **Mohammad J. Nasiri:** Writing – review & editing, Writing – original draft, Visualization, Software, Methodology, Investigation, Funding acquisition, Formal analysis, Conceptualization. **Vishwanath Venketaraman:** Writing – review & editing, Writing – original draft, Supervision, Conceptualization.

## Data availability statement

No new data were created or analyzed in this study. Data sharing is not applicable to this article.

## Funding

We appreciate the funding support from the NIH-10.13039/100000050NHLBI (2R15HL143545-02). MJN received financial support from the Research Department of the 10.13039/501100005851Shahid Beheshti University of Medical Sciences School of Medicine, Tehran, Iran (Grant no. 43014982).

## Declaration of competing interest

The authors declare that they have no known competing financial interests or personal relationships that could have appeared to influence the work reported in this paper.
